# Mutations in the *WTX *- gene are found in some high-grade microsatellite instable (MSI-H) colorectal cancers

**DOI:** 10.1186/1471-2407-10-413

**Published:** 2010-08-09

**Authors:** Silvio K Scheel, Marc Porzner, Sabine Pfeiffer, Steffen Ormanns, Thomas Kirchner, Andreas Jung

**Affiliations:** 1 Pathologisches Institut der Ludwig-Maximilians-Universität München, Thalkirchner Straße 36, 80337 Munich, Germany; 2 Universitätsklinik und Poliklinik für Innere Medizin I, Medizinische Fakultät der Martin-Luther Universität Halle-Wittenberg, Ernst-Grube Straße 40, 06097 Halle, Germany

## Abstract

**Background:**

Genetically, colorectal cancers (CRCs) can be subdivided into tumors with chromosomal instability (CIN) or microsatellite instability (MSI). In both types of CRCs genes that are involved in the degradation of β-CATENIN are frequently mutated. Whereas in CIN CRCs *APC *(Adenomatous Polyposis Coli) is affected in most cases, high grade MSI (MSI-H) CRCs frequently display mutations in various genes, like the *APC*-, *AXIN2*- or *CTNNBI *(β-CATENIN) gene itself. Recently in Wilms tumors, *WTX *(Wilms tumor gene on the X-chromosome) was discovered as another gene involved in the destruction of β-CATENIN. As the *WTX*-gene harbors a short T_6_-microsatellite in its N-terminal coding region, we hypothesized that frameshift-mutations might occur in MSI-H CRCs in the *WTX *gene, thus additionally contributing to the stabilization of β-CATENIN in human CRCs.

**Methods:**

DNA was extracted from 632 formalin-fixed, paraffin-embedded metastatic CRCs (UICCIV) and analyzed for MSI-H by investigating the stability of the highly sensitive microsatellite markers BAT25 and BAT26 applying fluorescence capillary electrophoresis (FCE). Then, in the MSI-H cases, well described mutational hot spot regions from the *APC*-, *AXIN2*- and *CTNNBI *genes were analyzed for genomic alterations by didesoxy-sequencing while the *WTX *T_6_-microsatellite was analyzed by fragment analysis. Additionally, the PCR products of T_5_-repeats were subcloned and mutations were validated using didesoxy-sequencing. Furthermore, the *KRAS *and the *BRAF *proto-oncogenes were analyzed for the most common activating mutations applying pyro-sequencing. mRNA expression of *WTX *from MSI-H and MSS cases and a panel of colorectal cancer cell lines was investigated using reverse transcription (RT-) PCR and FCE.

**Results:**

In our cohort of 632 metastatic CRCs (UICCIV) we identified 41 MSI-H cases (6.5%). Two of the 41 MSI-H cases (4.8%) displayed a frameshift mutation in the T_6_-repeat resulting in a T_5 _sequence. Only one case, a male patient, expressed the mutated *WTX *gene while being wild type for all other investigated genes.

**Conclusion:**

Mutations in the *WTX*-gene might compromise the function of the β-CATENIN destruction complex in only a small fraction of MSI-H CRCs thus contributing to the process of carcinogenesis.

## Background

Genetically, colorectal cancers (CRCs) might be subdivided into two groups. One group is characterized by chromosomal instability (CIN) and follows the classical multistep carcinogenesis model where mutations result in the activation of proto-oncogenes (gain of function) or the inhibition of tumor suppressor genes (loss of function) by this driving the process of colorectal carcinogenesis [1]. The other group is characterized by high grade instability of microsatellites (MSI-H) and can be subdivided into sporadic and heritable forms and accounts for approximately 15% of all CRCs. The majority of sporadic MSI-H CRCs is characterized by loss of expression of the *MLH1 *(MUT-L homologue 1) gene, a component of the mismatch repair (MMR) system due to methylation of its promoter/exon 1 region. These MSI-H CRCs belong to the CIMP (CpG island methylator phenotype) and are highly associated with mutations in the *BRAF *proto-oncogene (up to 75%) [2]. In contrast, the heritable forms of MSI-H CRCs, known as hereditary non polyposis colorectal cancers (HNPCC), harbor mutations in genes of the MMR-system, like *MLH1*, *MSH2 *(MUT-S homologue 2), *MSH6 *or *PMS2 *(post mitotic segregation 2). Thus, in MSI-H tumors, the function of the MMR-system is lost [3]. This in turn leads to frameshift mutations in microsatellites, which might contribute to the malignant transformation of tumor cells when located in the coding sequences of tumor suppressor genes like the *TGFBR2 *(TGF-β receptor type 2) [4]. This type of mutation and the associated occurrence of neoantigenic structures might explain why sporadic MSI-H CRCs have a better prognosis than microsatellite stable (MSS) CRCs [5].

Interestingly, the stabilization of β-CATENIN, which is the executor of the canonical WNT-signaling pathway, is affected in both, MSI-H and MSS CRCs. In the WT situation β-CATENIN is earmarked for degradation by a multi-protein complex assembled of at least APC, AXIN2, PP2A (pyro-phosphatase 2 A) and GSK3B (glycogen synthase kinase 3β). In MSS CRCs, the stabilization of β-CATENIN is mostly achieved by mutations in the tumor suppressor gene *APC*, which is considered to be the gatekeeper of colorectal carcinogenesis [1]. In contrast in MSI-H CRCs, the stabilization of β-CATENIN seems to be a later event and is achieved by loss of function in *APC *[6-8] in only 14-56%, while in 24% mutations are found in the *AXIN2*- [8] or in up to 43% in the *CTNNBI*-gene itself, depending on if investigating sporadic or heritable cases of CRC [6,8-11]. Expectedly in MSI-H CRCs, the loss of function mutations in the *APC*- and *AXIN2*-tumor suppressor genes partly results from frameshift mutations, thus highlighting the causative role of the MMR system in MSI-H CRCs.

Recently in Wilms tumors, *WTX *(Wilms Tumor gene on the X-Chromosome) was discovered as another component of the β-CATENIN degradation complex where it directly interacts with β-CATENIN and APC [12,13]. Moreover, Wilms tumors are often characterized by a deregulated WNT-signaling pathway which can be attributed to mutations in the *WTX*-gene in 7 to 30% of all cases [14,15]. Due to its location on the X-chromosome, *WTX *resembles a one hit tumor suppressor which has therefore a higher penetrance in men than in women.

Because of its role in the degradation of β-CATENIN and the presence of a short six basepair long T-repeat (T_6_) in the N-terminus encoding part of the *WTX*-gene, we hypothesized that *WTX *might also contribute to the stabilization of β-CATENIN in MSI-H CRCs. Therefore, we investigated if frameshift mutations are present in the T_6_-repeat of the *WTX*-gene in the MSI-H fraction of a collection of metastatic CRCs (UICCIV).

## Methods

### Clinical samples

632 cases of formalin fixed, paraffin-embedded (FFPE) tissue of metastatic CRCs (UICCIV) were selected for the investigation. The usage of these cases for scientific reasons was approved by the local ethics committee of the Medical Faculty of the Ludwig-Maximilians-Universität München.

### DNA and RNA isolation

After removal of paraffin wax using graded xylene- and alcohol dilutions following routine protocols, tumor cells were manually dissected from the slides. Subsequently, DNA and RNA were extracted using QIAamp DNA Micro kits or RNeasy FFPE kits (Qiagen, Hilden, Germany), respectively following the instruction manuals.

### Cell lines, Cell culture and RNA isolation

Cultivated colorectal tumor cell lines RKO, LoVo, SW480, Caco2, DLD-1, HCT15, HCT116, LS174T and HT29 were purchased from the ATCC (LGL Promochem GmbH, Wesel, Germany) and maintained in DMEM (Biochrom, Berlin, Germany) containing 7.5% (v/v) fetal bovine serum (Biochrom, Berlin, Gemany). RNA was isolated using RNeasy Mini kits (Qiagen, Hilden, Germany) according to the user's instructions.

### PCR, reverse transcription, RT-PCR, fluorescence capillary electrophoresis (FCE), didesoxy- and pyro-sequencing

For the determination of MSI-H polymerase chain reactions (PCRs) specific for the monomorphic mononucleotide microsatellites *BAT25 *and *BAT26 *were done using 1 μl DNA as the template in the presence of 1.5 mM MgCl_2_, 200 μM dNTPs (Fermentas, St. Leon, Germany), 400 nM of each of *BAT25 *or *BAT26 *specific primer pairs (Additional file 1, Table S1), respectively together with 1 U HotStarTaq Polymerase (Qiagen, Hilden, Germany) following the user's manual. Male DNA (Promega, Mannheim, Germany) was used as the template for positive- and water for negative controls. PCRs specific for the mutation hot spots of the *APC*- (exon 15) [7], *AXIN2*- (exon 8) [8] and *CTNNBI*- (exon 3) [16,17] genes as well as the T_6_-microsatellite of the *WTX*-gene were done using the same protocol together with gene-specific primers (Additional file 1, Table S1). 1 μl of the final PCR products was used for subsequent sequencing using BigDye Terminator v1.1 kits (Applied Biosystems, Darmstadt, Germany) together with appropriate primers (500 nM, Additional file 1, Table S1). Reactions were purified using DyeEx v2.0 kits (Qiagen, Hilden, Germany) following the handbook. 4 μl purified PCR product were mixed together with 16 μl highly-deionized formamide (HiDi, Applied Biosystems, Darmstadt, Germany), heated for 2 minutes at 90°C, cooled down immediately on ice and loaded onto the genetic analyzer 3130 (Applied Biosystems, Darmstadt, Germany). Results were finally analyzed with the help of the Sequencing Analysis v5.2- (Applied Biosystems, Darmstadt, Germany) and Geneious-software (Biomatters Ltd., Australia).

**Table 1:** Overview of patient age, gender and mutations found in MSI-H CRCs

Patient ID	Age	Gender	*WTX*	*APC* *Exon 15*	*AXIN2* *Exon 8*	*CTNNB1* *Exon 3*	*KRAS* *Codon 12/13*	*BRAF* *Codon 600*
1	65	m	T_5_	WT	WT	WT	WT	WT

2	n.a.	f	T_5_	WT	WT	WT	WT	c.1799T>A

3	52	m	T_6_	WT	WT	WT	WT	WT

4	74	m	T_6_	WT	1 bp del (G) codon 665	WT	WT	n.a.

5	68	f	T_6_	WT	WT	WT	c.35G>A	WT

6	28	m	T_6_	2 bp del (AG) codon 1462	WT	WT	WT	WT

7	72	f	T_6_	WT	WT	WT	WT	c.1799T>A

8	65	f	T_6_	n.a.	n.a.	n.a.	WT	WT

9	63	f	T_6_	c.2312A>G p.E770G	WT	WT	WT	c.1799T>A

10	n.a.	m	T_6_	c.2393A>G p.G797D	WT	WT	WT	c.1799T>A

11	77	f	T_6_	WT	WT	WT	WT	WT

12	65	f	T_6_	WT	WT	WT	WT	c.1799T>A

13	75	m	T_6_	1 bp ins (A) codon 1554	WT	WT	WT	c.1799T>A

14	69	f	T_6_	WT	c.2062C>T (mut/mut) p.P687L	c.64G>A p.V22I	WT	WT

15	72	f	T_6_	WT	WT	WT	WT	c.1799T>A

16	46	m	T_6_	WT	c.2073C>T p.691Q>STOP	WT	WT	WT

17	45	f	T_6_	1 bp ins (A) codon 1554	WT	WT	WT	c.1799T>A

18	42	m	T_6_	WT	WT	WT	WT	WT

19	n.a.	m	T_6_	WT	WT	WT	WT	WT

20	76	m	T_6_	WT	WT	WT	WT	c.1799T>A

21	76	m	T_6_	WT	WT	WT	WT	WT

22	78	f	T_6_	WT	c.2037C>T p.H679Y and c.2071C>T p.691Q>STOP	WT	WT	WT

23	56	f	T_6_	WT	WT	c.134C>T p.S45F	WT	c.1799T>A

24	33	m	T_6_	WT	WT	WT	WT	WT

25	73	f	T_6_	WT	WT	WT	WT	c.1799T>A

26	65	f	T_6_	WT	c.2103T>C p.S701P	WT	WT	c.1799T>A

27	77	f	T_6_	WT	WT	WT	WT	WT

28	69	m	T_6_	WT	WT	WT	WT	WT

29	43	m	T_6_	WT	WT	WT	WT	WT

30	64	f	T_6_	WT	WT	WT	WT	WT

31	43	m	T_6_	WT	WT	WT	WT	WT

32	43	m	T_6_	WT	WT	WT	WT	WT

33	54	m	T_6_	WT	WT	WT	WT	WT

34	66	f	T_6_	WT	c.2062C>T p.P687L	WT	WT	WT

35	40	f	T_6_	WT	WT	WT	WT	WT

36	60	m	T_6_	n.a.	n.a.	n.a.	n.a.	WT

37	63	f	T_6_	WT	WT	WT	c.35G>A	WT

38	73	m	T_6_	WT	WT	WT	WT	WT

39	72	f	T_6_	WT	c.2077A>G p.H692R	WT	WT	WT

40	65	f	T_6_	n.a.	n.a.	n.a.	WT	n.a.

41	52	m	T_6_	c.2626C>T p.876R>STOP and 2 bp del (AG) codon 1462	WT	WT	WT	WT

For sequencing the fragments generated from the WTX-gene, PCRs were repeated using the same but unlabelled primers (Additional file 1, Table S1) as for the screening for mutations. Resulting PCR products were subcloned using the CloneJet PCR cloning kit (Fermentas, St. Leon, Germany) according to the user's manual and finally sequenced as described above.

For reverse transcription, 1 μg of RNA was converted into cDNA using QuantiTect Reverse Transcription kits (Qiagen, Hilden, Germany) in the presence (+) or absence (-) of reverse transcriptase (RT) following the user's manual. For subsequent *WTX *specific PCRs 1 μl of a fiftyfold diluted cDNA solution was used as the template but else applying the same conditions as described above. RT-PCR specific for the housekeeping gene *ACTB *(β-ACTIN, [GenBank: NM_001101]) served as a control using β-ACTIN specific primers (Additional file 1, Table S1). Products from RT-PCR were separated on 2% 0.5 × TBE agarose gels containing 100 ng/ml ethidium bromide and visualized under UV-light.

For the analysis microsatellite-stability PCR products were separated by capillary electrophoresis using an ABI 3130 sequencing analyzer by mixing 1 μl of the PCR products with 18.5 μl highly-deionized formamide (HiDi) and 0.5 μl of the GeneScan-500 LIZ size standard (both Applied Biosystems, Darmstadt, Germany), heated for 2 minutes at 90°C, cooled down immediately on ice, and subsequently loaded onto the genetic analyzer 3130. Finally, results were analyzed with the help of the GeneMapper v4.0 software (Applied Biosystems, Darmstadt, Germany).

Mutations in codons 12 and 13 of the *KRAS- *and codon 600 of the *BRAF*-gene were done as described [18,19] using Pyromark-Gold reagents (Qiagen, Hilden Germany) together with the appropriate primers (Additional file 1, Table S1) following the user's instructions.

### Results

**Figure 1:**
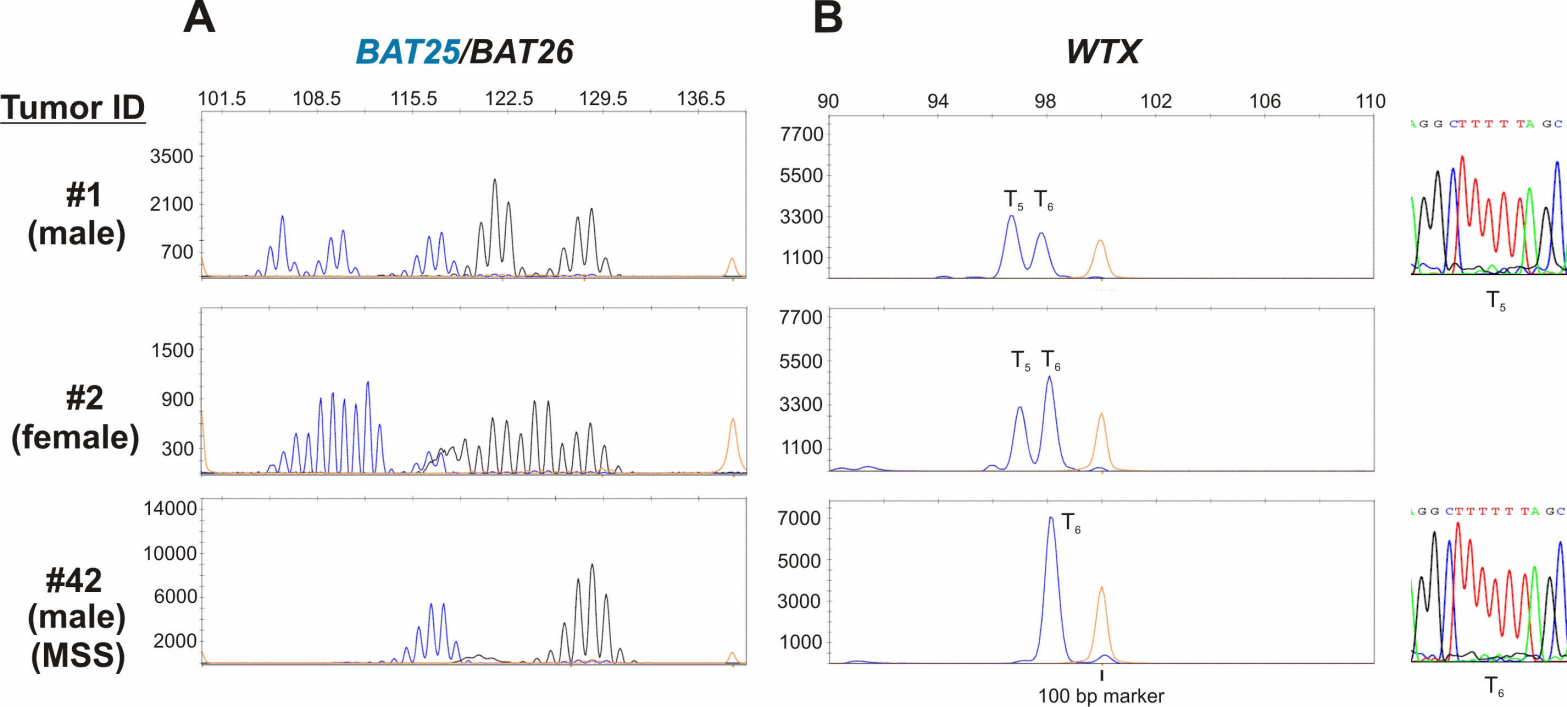
**MSI-H colorectal tumors display frameshift mutations in the T_6_-microsatellite of the *WTX*-gene**. (A) Genomic DNA from 632 patients with metastatic CRC (UICCIV) was analyzed for the presence of MSI-H with the help of the two monomorphic microsatellite markers *BAT25 *and *BAT26*. Shown are representative results from two CRCs with MSI-H (ID #1, #2) and one CRC with MSS (ID #42). (B) Two tumors of the 41 MSI-H cases of CRCs harbored frameshift mutations in the coding sequence of the T_6_-repeat of the *WTX*-gene resulting in the occurrence of a T_5_-repeat (ID #1 and #2) compared to only wild type T_6_-repeats in tumors with MSS CRCs (ID #3). Left: shown by FCE, right: shown by direct sequencing.

**Figure 2:**
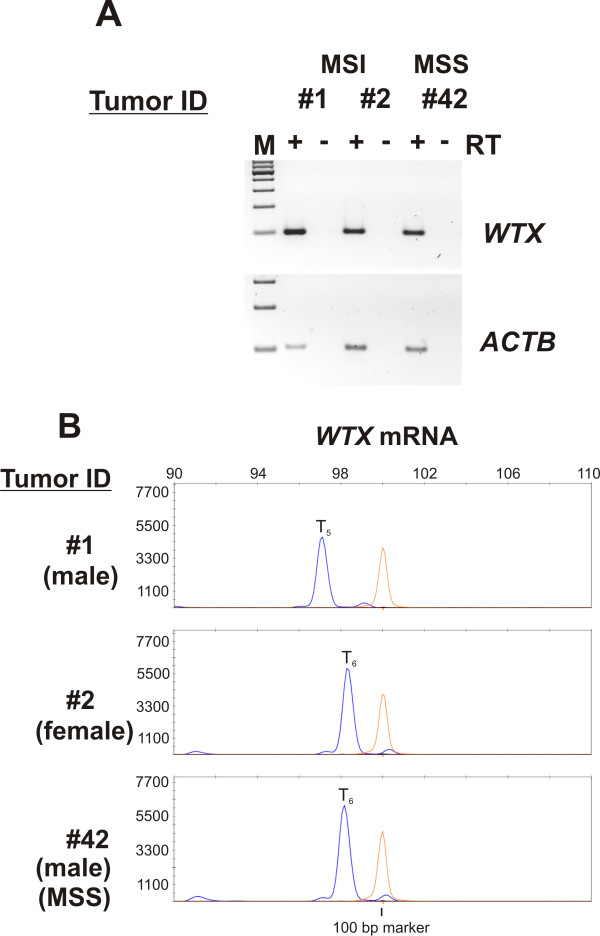
**Frameshift mutations in the *WTX*-gene are expressed on the mRNA level**. (A) CRCs (ID #1, #2 and #42) expressed *WTX *mRNA. (B) One of the two CRCs with MSI-H and a genomic frameshift mutation in the *WTX*-gene displayed expression of the mutated allele on the level of mRNA (ID #1) whereas the other tumor (ID #2) as well as MSS-CRCs (ID #42) harbored wild type mRNA indicated by the presence of only T_6_-repeats.

First of all, MSI-H CRCs were identified from our collection of 632 cases of metastatic CRCs (UICCIV) by investigating the stability of the highly sensitive and specific monomorphic mononucleotide markers *BAT25 *and *BAT26 *[20,21] using PCR based fluorescence capillary electrophoresis (FCE). We detected instability of both microsatellite markers in 41 out of 632 cases (6.5%) compared to control DNA (Figure 1A, tumor ID #1, #2 (both MSI-H), #42 (MSS) and Table 1). These 41 cases were taken for all further investigations as we aimed to concentrate on *WTX *frameshift mutations only in MSI-H CRCs. Second, these cases were analyzed for mutations in the *BRAF- *and *KRAS*- proto-oncogenes (Table 1) applying pyro-sequencing. We found two out of 40 evaluable cases (5%) with activating mutations in *KRAS *codons12 and 13 and 12 out of 39 cases (30.8%) with an activating mutation in codon 600 of the *BRAF *gene (p.V600E). Third, *WTX *frameshift mutations were found in 2 out of the 41 investigated MSI-H CRCs (4.9%) as shown by FCE and resulted in a T_5_-repeat (Figure 1B, tumor ID #1, #2), while no such mutations were present in MSS CRCs (Figure 1B, ID #42). These findings were further confirmed by subcloning the truncated *WTX *PCR products and subsequent didesoxy-sequencing. Both approaches also revealed signals from the wild type T_6_-repeat in both cases (not shown for direct sequencing), which probably stem from contaminations with normal tissue, as well as signals derived from the second, intact X-chromosome, the latter of course only in case of female patients. The detected frameshift leads to a stop codon (TGA) at position 621 of the coding sequence of the *WTX*-gene, which is of 3.408 bp in length (GenBank: NM_152424). When expressed, such mutated *WTX *alleles will inevitably result in a truncated protein of 207 instead of wild typic 1.135 amino acids. Such a protein will have no functional properties with respect to the degradation of β-CATENIN, since the APC and β-CATENIN interaction domains are located C-terminally of amino acid 207, namely positions 307-789 and in the C-terminus, respectively [12,13]. Tumor ID #2 was additionally characterized by a mutation in the *BRAF *gene (Table 1). Fourth, we wanted to increase the evidence that the *WTX *mutations might have a functional relevance for the stabilization of β-CATENIN in the context of the other members of the WNT signaling pathway *APC*, *AXIN2 *and *CTNNB1*, which have been described to be mutated in MSI-H CRCs. Therefore, we investigated the described mutational hot spot regions of these three genes in the 41 MSI-H CRCs [7,8,16,17] by direct didesoxy-sequencing (Table 1). Mutations in the APC gene were detected in 6 out of 38 evaluable cases (15.8%), in the *AXIN2*-gene in 7 of 38 (18.4%) and in the *CTNNBI*-gene in 2 of 38 (5.3%) cases. As far as to our knowledge, some of the detected mutations have not been described previously and thus their functional consequences remain to be determined. Importantly, however, we could not find any additional mutations in the two tumors displaying the *WTX *T_5 _frameshift. Fifth, we analyzed the expression of *WTX *mRNAs in the tumors of the patients with *WTX *frameshift mutations in order to assess whether these might be of functional relevance for the stabilization of β-CATENIN. Due to the clonal origin of cancers and the fact that only one X-chromosome is transcriptionally active in women whereas men carry only a single X-chromosome, mutations in X-chromosomal encoded genes like the *WTX*-gene will have a direct functional consequence known as haplo-insufficiency when being expressed. Thus, we checked, first of all, if WTX is commonly expressed in CRCs and especially in the patients carrying T_5_-repeats in their WTX-genes. The tumors of both patients expressed *WTX *mRNA (Figure 2A, tumor ID #1 and #2), as did MSI-H tumors from patients with wild type *WTX *genes (Additional file 2, Figure S1 A patient ID #4 - #6) or tumors from MSS CRCs (Figure 2A, tumor ID #42 and Additional file 2, Figure S1 A, tumor ID #43 and #44), as well as a panel of cultivated colorectal tumor cell lines (Additional file 2, Figure S1 B). Next, we investigated the length of the T_6_- microsatellite in the *WTX*-mRNA. We found that only one of the two tumors (ID #1, a male patient) harbored the T_5_-frameshift mutation on the transcript level (Figure 2B, tumor ID #1) and thus inevitably also on the protein level. However, this latter fact could not be verified immunohistochemically due to lack of specific antibodies distinguishing between wild type and mutated WTX protein. The T_5_-repeat containing tumor of the female patient (ID #2) expressed wild-type *WTX*-mRNA, indicating that the mutated gene was located on the inactivated X-chromosome.

## Discussion

MSI-H CRCs constitute approximately 15% of all CRCs [3] and are generally considered to be characterized by a better prognosis than tumors of the MSS type [5]. This is reflected by the fact that only 7.9% of all MSI-H CRCs show progression to a metastatic state (UICCIV), while 27.9% of MSS cases succeed in forming distant metastases [22]. Thus, the proportion of MSI-H CRCs among tumors in UICCIV has been found to be less compared with the mean value of 15% in all stages (UICCI - UICCIV) [23] which is in support with 41 MSI-H cases found in 632 UICCIV cases of CRCs (6.5%). These results were obtained using the two mononucleotide markers *BAT25 *and *BAT26 *instead of the five marker set recommended by the Bethesda guidelines from the National Cancer Institute [20] because these two markers were shown to be of almost the same diagnostic value as all five NCI markers in combination [21]. Furthermore, due to their monomorphic character, usage of *BAT25 *and *BAT26 *makes a comparison of normal tissue dispensable.

The difference in clinical behavior of colorectal MSI-H and MSS tumors is also reflected by differences in the mechanisms of genetic instability. MSS CRCs usually mutate the *APC *tumor suppressor gene by gross deletions in the genome known as loss of heterozygosity (LOH) or point mutations [24]. As this occurs as the entry mutation in the process of colorectal carcinogenesis *APC *is here known as the gatekeeper [1]. In contrast, MSI-H CRCs seem to evolve via a different pathway as mutations in the *BRAF *or less often *KRAS *proto-oncogene occur early [3]. Our findings show that in UICCIV less tumors carry *BRAF *gene mutations (28% compared with up to 75% in MSI-H tumors of all stages [2]) indicating that in MSI-H tumors *BRAF *mutations might be somehow protective for progression into UICCIV. Alternatively, the amount of heritable HNPCC tumors might be higher in our collection which do not show mutations in the *BRAF *gene [2]. Due to changes in the cellular methylation system in sporadic MSI-H tumors the expression of several tumor suppressor genes is turned off due to methylation of its promoter/exon1. This also seems to be the cause for the loss of the expression of the *MLH1 *(*MUT-L homologue*) gene. As a consequence, instability of microsatellites occurs which affects in a second wave of mutations components of the WNT-signaling pathway, like the *APC*-, or the *AXIN2*-gene and, as shown here in a small group of cases, additionally in the *WTX*-gene. Since we concentrated on defects leading to the stabilization of β-CATENIN in MSI-H CRCs, a collection of UICCIV tumors was used because it was expected that later states should contain most if not all mutations leading to the stabilization of β-CATENIN. One study analyzed in a group of 45 MSI-H CRCs the occurrence of mutations in the three genes encoding *AXIN2 *(11/45 - 24.4%), *APC *(4/28 - 14.3%) and *CTNNBI *(5/45 - 11.1%) [8], thus assigning approximately 50% of MSI-H CRCs with defects in components of the WNT signaling pathway. Assuming that mutations in the WNT-signaling pathway might also be important in MSI-H tumors, it suggests that additional mechanisms might contribute to the stabilization of β-CATENIN in this tumor entity. The situation turns out to be even more complex as heritable and sporadic forms of tumors display partly great differences in their mutational spectrum as e.g. shown for the *CTNNB1 *gene where sporadic tumors do not display mutation [11] compared to 43% in heritable HNPCC cases [10]. Analyzing our mixed collection of UICCIV MSI-H CRCs, we found 15 out 38 evaluable cases (39.5%) with mutations in the established components of the WNT signaling pathway. Alternatively, this might be a stage specific effect as it was shown that MSI-H tumors are found less frequently in the UICCIV group than MSS tumors which is not the case in the other stages (UICCI - UICCIII) thus indicating a special biology of MSI-H tumors in UICCIV [22]. Here, we add frameshift mutations in the recently identified *WTX*-gene, a component of the β-CATENIN degradation complex [13], as an additional mechanism which were found at a frequency of 2/41 among MSI-H cases (4.9%). This frameshift occurred in a 6 thymidine repeat that is located in the N-terminal coding region giving rise to an unfunctional gene product when being transcribed as shown here and translated due to the absence of the APC- and β-CATENIN interaction domains [12,13]. Moreover, we detected several monorepeats of five basepairs in length distributed over the *WTX *open reading frame which we did not investigate for instability because they are to small to be targets for microsatellite frameshifts [25].

In general a rate of two out of 41 is very low but it is misleading to conclude from mutation rates on the importance of genes as even intronic regions display mutation rates of 54.2% which are known to be only passenger mutations [26]. Moreover, even high mutation frequencies of 39% in the *TCF4 *gene (T-cell transcription factor 4) [27] which has an important role in WNT-signaling are not warranting that the mutations might have a physiologic effect [28]. Thus, the finding that 1 patient (2.5%) expressed the genetic alteration on the mRNA level may be an indicator that alterations in the *WTX*-gene might have a functional role for the stabilization of β-CATENIN and have in this case the status of a driver mutation. This accounts especially in the context of absent other mutations in the WNT pathway (Table 1) and when considering that this patient with tumor ID #1 was a 65 years old man with probably a sporadic tumor and that *WTX *is located on the X-chromosome. Moreover, our mutation frequency is consistent with another study, which detected only a single mutation in 47 CRCs (2.1%), irrespective of the MSI status [29]. While the functional consequene of this described point mutation is unknown, this and our work at least indicate that mutations in *WTX *might be involved in colorectal carcinogenesis in a small proportion of both, MSI-H as well as MSS tumors. Interestingly, our result that the tumor of a female patient displayed the mutation in the *WTX*-gene on the transcriptionally inactive X-chromosome is in support with findings from Wilms tumors where mutations of the *WTX*-gene were also found on the inactivated X-chromosome in women [14]. Obviously, larger cohorts are needed for definitively answering the open questions about the frequency of *WTX*-gene mutations and the prevalence of mutations on the inactivated X-chromosome in female patients.

## Conclusion

Taking together, we demonstrate that mutations in the *WTX*-gene might play a role in the process of MSI-H colorectal carcinogenesis in a small subgroup of these tumors as has been modeled for Wilms Tumors before [30]. This implies that *WTX*-gene mutations should be reconsidered in future studies dealing with MSI-H CRCs, especially in the context of the other known mutations leading to stabilization of β-CATENIN. Importantly, a functional role of these mutations for the process of colorectal carcinogenesis has to be investigated in further works in the future.

## Competing interests

The authors declare that they have no competing interests.

## Authors' contributions

SKS coordinated the study, designed and partly optimized the analytical tools. He generated and analyzed the data. Moreover, he wrote the draft of the manuscript. SP generated and analyzed data in part, MP developed and optimized part of the mutation detection system of the APC gene as part of his MD work, SO was involved in the initial planning of the study, TK approved the study and AJ designed and coordinated the study. He was involved in the analysis of the data and brought the manuscript into its final form. All authors read and approved to the final form of the manuscript.

## Author's information

This project is part of the PhD thesis of SKS.

## Pre-publication history

The pre-publication history for this paper can be accessed here:

http://www.biomedcentral.com/1471-2407/10/413/prepub

## Supplementary Material

Additional file 1 Table S1 Oligonucleotides used in this study. Click here for file

Additional file 2 **Figure S1 CRCs as well as colorectal tumor cell lines express *WTX *mRNA**. (A) All cases of CRCs tested expressed *WTX *mRNA irrespective of being MSI-H (ID #1, #2 and #4 to #6) or MSS (ID #42 to #44). (B) A panel of cultivated CRC cell lines also expressed *WTX *mRNA. PCR specific for the housekeeping gene *ACTB *(β-ACTIN) served as a control. Click here for file
