# Supplementary material for: Mutations in the WTX - gene are found in some high-grade microsatellite instable (MSI-H) colorectal cancers
Source: BMC Cancer. 2010 Aug 9;10:413. doi: 10.1186/1471-2407-10-413 (PMC2928794; doi:10.1186/1471-2407-10-413)
Supplement: Additional file 1 — Table S1 Oligonucleotides used in this study. [file 1471-2407-10-413-S2.DOC]

**Additional Table S1:** Oligonucleotides used in this study

| **Primer** |  | **Sequence** |
| --- | --- | --- |

| **Analysis of microsatellite instability** | | |
| --- | --- | --- |
| ***BAT25*** | F  R | TCGCCTCCAAGAATGTAAGT-FAM  TCTGCATTTTAACTATGGCTC |
| ***BAT26*** | F  R | TGACTACTTTTGACTTCAGCC-TET  AACCATTCAACATTTTTAACCC |
| ***WTX*** | F R | GCTGTGGCTGAGAAGTTTCC-FAM  CCCCAGTGACCTTGCTCTT |

| **Analysis of mutations** | | |
| --- | --- | --- |
| ***APC I***  ***APC I N*** | F  R  FN  RN  SF  SR | GCTCAAGCTTGCCATCTCTT  TGAAGAGGAGCTGGGTAACA  GTAAAACGACGGCCAGTGCTCAAGCTTGCCATCTCTTCATG *TAATACGACTCACTATAGG*TGAAGAGGAGCTGGGTAACACTGT  UNI  T7 |
| ***APC II***  ***APC II N*** | F  R  FN  RN  SF  SR | CTGGCAACATGACTGTCCTT  TGAGAGGTATGAATGGCTGACA  GTAAAACGACGGCCAGTCTGGCAACATGACTGTCCTTTCAC *TAATACGACTCACTATAGG*TGAGAGGTATGAATGGCTGACACTT  UNI  T7 |
| ***APC III***  ***APC III N*** | F  R  FN  RN  SF  SR | TTTGCAGATCTCCACCACTG  TTTGGCATAAGGCATAGAACA  GTAAAACGACGGCCAGTTTTGCAGATCTCCACCACTGCAG *TAATACGACTCACTATAGG*TTTGGCATAAGGCATAGAACATGTCCTA  UNI  T7 |
| ***APC IV***  ***APC IV N*** | F  R  FN  RN  SF  SR | AAGAAGCTCTGCTGCCCATA  TAGGTCGGCTGGGTATTGAC  GTAAAACGACGGCCAGTAAGAAGCTCTGCTGCCCATACACA  *TAATACGACTCACTATAGG*TAGGTCGGCTGGGTATTGACCATA  UNI  T7 |
| ***APC 1***  ***APC 1 N*** | F  R  FN  RN  SF  SR | CAGATTCTGCTAATACCCTGC  CTAAACATGAGTGGGGTCTC  CAGATTCTGCTAATACCCTGCAAATAGCAG  CTAAACATGAGTGGGGTCTCCTGAAC  CAGATTCTGCTAATACCCTGCAAATAGCAGAAAT  TAAACATGAGTGGGGTCTCCTGAACATAG |
| ***APC 2***  ***APC 2 N*** | F  R  FN RN  SF  SR | CTTCAGGAGCGAAATCTCC  AGGTGGTGGAGGTGTTTTAC  TTCAGGAGCGAAATCTCCCTCC  AGGTGGTGGAGGTGTTTTACTTC  CAGGAGCGAAATCTCCCTCCAAAAG  AGGTGGTGGAGGTGTTTTACTTCTGCT |
| ***APC 3***  ***APC 3 N*** | F  R  FN  RN  SF  SR | CCCAGTGATCTTCCAGATAG  CAAGAAAATCCATCTGGAGTAC  CCCAGTGATCTTCCAGATAGCCC  CAAGAAAATCCATCTGGAGTACTTTCCG  CAGTGATCTTCCAGATAGCCCTGGA  AAGAAAATCCATCTGGAGTACTTTCCGTGG |
| ***APC 4***  ***APC 4 N*** | F  R  FN RN  SF  SR | GGTTCTTCCAGATGCTGATAC  TAATAGGTCCTTTTCAGAATCAATAG  GGTTCTTCCAGATGCTGATACTTTATTAC  TAATAGGTCCTTTTCAGAATCAATAGTTTTTTCTG  GGTTCTTCCAGATGCTGATACTTTATTACATT  TAATAGGTCCTTTTCAGAATCAATAGTTTTTTCTGCCT |
| ***APC 5***  ***APC 5 N*** | F  R  FN  RN  SF  SR | CAGAATCAGAGCAGCCTAAAG  TTTGTACACAGGCAGCTGAC  CAGAATCAGAGCAGCCTAAAGAATC  TTTGTACACAGGCAGCTGACTTG  CAGAATCAGAGCAGCCTAAAGAATCAAATG  TTTGTACACAGGCAGCTGACTTGGTTTC |
| ***Axin2***  ***Axin2 N*** | F  R  FN  RN  SF  SR | CCTACCCCTTGGAGTCTGC  GGACCCTTCACTTCCACTCA  GTAAAACGACGGCCAGTCCTACCCCTTGGAGTCTGC  *TAATACGACTCACTATAGG*GCCTCAACCTAGGACCCTTC  UNI  T7 |
| ***CTNNBI*** | F  R  FN  RN  SF  SR | CTGACTTTCAGTAAGGCAATG  TAATACTCTTACCAGCTACTTG  GTAAAACGACGGCCAGTGCCAATCTACTAATGCTAATACT  *TAATACGACTCACTATAGG*TAATACTCTTACCAGCTACTTG  UNI  T7 |
| ***UNI***  ***T7*** |  | GTAAAACGACGGCCAGT  TAATACGACTCACTATAGG |
| ***KRAS*** | F  R  S  F  R  S | NNNGGCCTGCTGAAAATGACTGAA  TTAGCTGTATCGTCAAGGCACTCT-BIO  TGTGGTAGTTGGAGCT  TGACTGAATATAAACTTGTGGTAGTTG-BIO  TCGTCCACAAAATGATTCTGA  GCACTCTTGCCTACG |
| ***BRAF*** | F  R  S | TGAAGACCTCACAGTAAAAATAGG  TCCAGACAACTGTTCAAACTGAT-BIO  GTAAAAATAGGTGATTTTGG |

| **Analysis of mRNA Expression** | | |
| --- | --- | --- |
| ***WTX*** | F  R | GCTGTGGCTGAGAAGTTTCC  CCCCAGTGACCTTGCTCTT |
| ***ACTB*** | F  R | TTGCGGATGTCCACGTCA  GCCCTGAGGCACTCTTCCA |

For the APC gene two mutation clustering regions have been described for MSI-H colorectal tumors. The first ranges from position 2,237 to 2,859 of the coding sequence (CDS) of the APC gene (accession number: M74088) the second from 3,887 to bp 4,848[1] which is commonly known as the mutation cluster region (MCR).[2] The first cluster is covered by overlapping fragments identified by the primer pairs of APCI to APCIV and the second cluster (MCR) by primer pairs APC1 to APC4, respectively. Some of the primer are modified by a tail which might be the sequence of the M13 phage universal (UNI,underlined) or T7-phage RNA promoter (T7, underlined italics).

Abbreviations: BIO – biotin label at the 5’ end of the primer, F – forward primer, FAM – 6-Carboxyfluorescein label at the 5’ end of the primer, FN – forward primer of nested reaction, N – nested, R – reverse primer, RN – reverse primer of nested direction, S – sequencing primer, SF – sequencing primer forward direction, SR – sequencing primer reverse direction, TET – 6-carboxy-1,4-dichloro-2’,7’-dichloro fluorescein label at the 5’ end of the primer, T7 – T7 page RNA promoter binding site, UNI – universal or M13 universal primer.

1. Huang J, Papadopoulos N, McKinley AJ, Farrington SM, Curtis LJ, Wyllie AH, Zheng S, Willson JK, Markowitz SD, Morin P, et al: **APC mutations in colorectal tumors with mismatch repair deficiency.** *Proc Natl Acad Sci U S A* 1996, **93:**9049-9054.

2. Bienz M: **The subcellular destinations of APC proteins.** *Nat Rev Mol Cell Biol* 2002, **3:**328-338.
